# *FKS1* mutation associated with decreased echinocandin susceptibility of *Aspergillus fumigatus* following anidulafungin exposure

**DOI:** 10.1038/s41598-020-68706-8

**Published:** 2020-07-20

**Authors:** Ana Pinto e Silva, Isabel Marcos Miranda, Joana Branco, Patricia Oliveira, Isabel Faria-Ramos, Raquel M. Silva, Acácio Gonçalves Rodrigues, Sofia Costa-de-Oliveira

**Affiliations:** 1 Division of Microbiology, Department of Pathology, Faculty of Medicine, University of Porto, Al. Hernâni Monteiro, 4200-319 Porto, Portugal; 2 Center for Research in Health Technologies and Information Systems (CINTESIS), R. Dr. Plácido da Costa, 4200-450 Porto, Portugal; 3 Department of Surgery and Physiology and Cardiovascular R&D Unit, Faculty of Medicine, University of Porto, Al. Hernâni Monteiro, 4200-319 Porto, Portugal; 4 Department of Medical Sciences, iBIMED and IEETA, University of Aveiro, Aveiro, Portugal; 5 Burn Unit, São João Hospital Center, Al. Hernâni Monteiro, 4200-319 Porto, Portugal

**Keywords:** Microbiology, Medical research

## Abstract

Invasive aspergillosis (IA) is a potentially lethal infection that affects mostly immunocompromised patients caused by *Aspergillus fumigatus*. Echinocandins are a second-line therapy against IA, used as a salvage therapy as well as for empirical or prophylactic therapy. Although they cause lysis of growing hyphal tips, they are considered fungistatic against molds. In vivo echinocandins resistance is uncommon; however, its wide clinical use could shortly lead to the emergence of *A. fumigatus* resistance. The aims of the present work was to assess the development of reduced echinocandins susceptibility phenotype by a *A. fumigatus* strain and to unveil the molecular mechanism underlying such phenotype. We induced in vitro cross-resistance to echinocandins following exposure of *A. fumigatus* to anidulafungin. Stability of the resistant phenotype was confirmed after removal of anidulafungin pressure. The *FKS1* gene was partially sequenced and a E671Q mutation was found. A computational approach suggests that it can play an important role in echinocandin resistance. Given the emerging importance of this mechanism for clinical resistance in pathogenic fungi, it would be prudent to be alert to the potential evolution of this resistant mechanism in *Aspergillus* spp infecting patients under echinocandins therapeutics.

## Introduction

Invasive aspergillosis (IA) is a potentially lethal infection afflicting mostly immunocompromised patients, the majority of cases caused by *Aspergillus fumigatus*. Early appropriate therapy is critical for the successful management. Echinocandins are clinically used in salvage therapy of IA as well as for empirical or prophylactic therapy^1,2^. Moreover, combination of voriconazole and anidulafungin (AFG) have been shown to be effective against azole-susceptible and azole-resistant *A. fumigatus* isolates^3^. The mechanism of action of echinocandins involves noncompetitive inhibition of (1,3)-β-D-glucan synthase, an essential enzyme involved in fungal cell wall synthesis. Echinocandins has been shown to cause lysis of growing hyphal tips but are considered fungistatic against moulds^4^. Elevated echinocandin Minimal Inhibitory Concentration (MIC) values for a variety of *Candida* clinical isolates were linked with genetic mutations in the hot spot regions of *FKS1* and *FKS2* genes^5,6^. Echinocandin resistance mechanisms are not yet clearly elucidated for *Aspergillus* spp. as in case of *Candida* spp.^7^.

The aim of the present work was to assess the development of reduced echinocandin susceptibility by an *A. fumigatus* clinical isolate exposed repeatedly in vitro to AFG and unveil the underlying molecular mechanisms.

## Results and discussion

**Figure 1:**
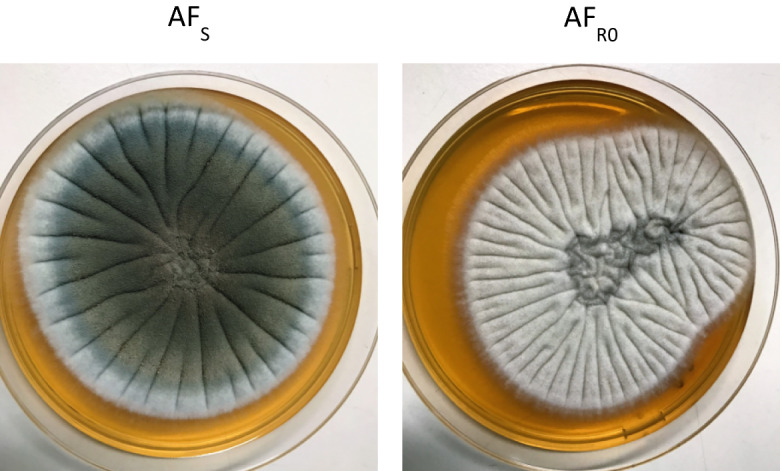
Photographs of YEPD agar plates showing *A. fumigatus* colony morphology following exposure to anidulafungin. AF_S_, initial susceptible strain. AF_R0_, resistant induced strain.

**Figure 2:**
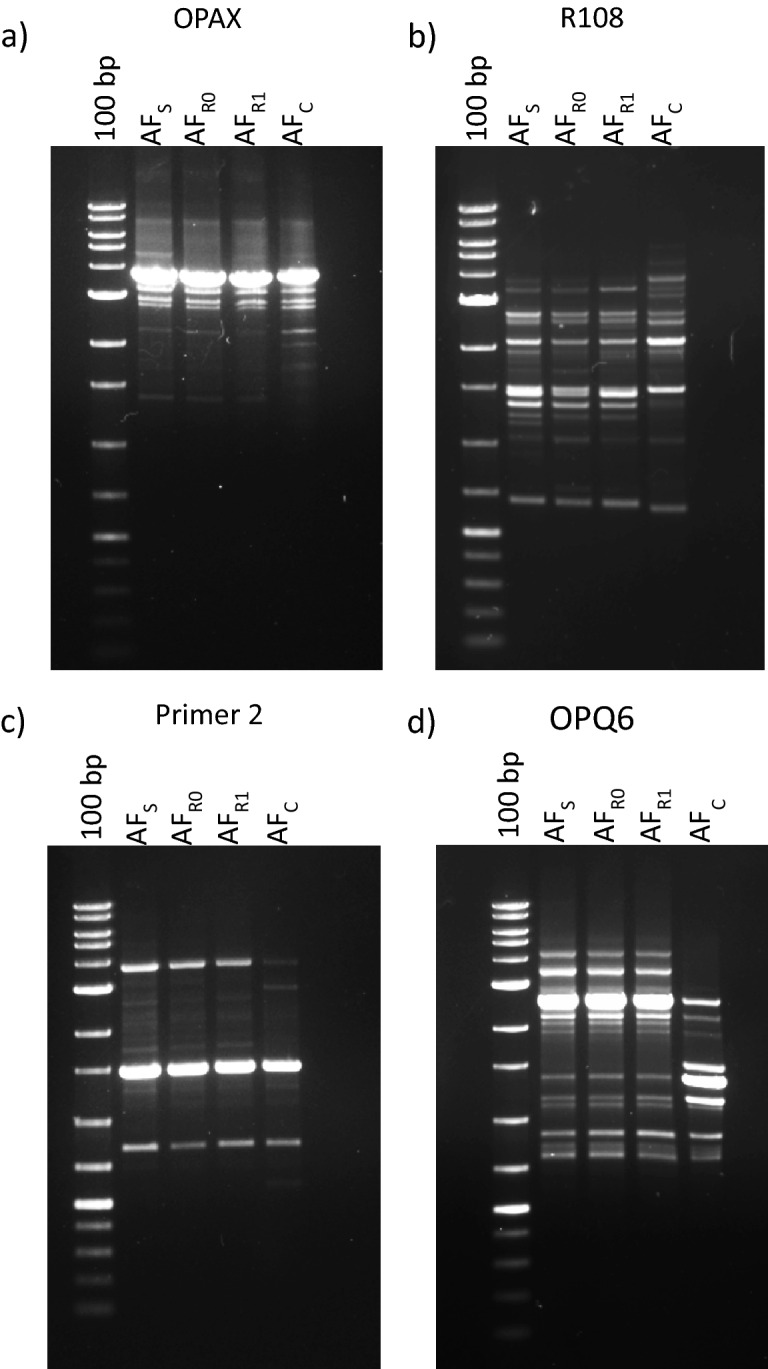
Random amplification of polymorphic DNA patterns, using primers (**a**) OPAX and (**b**) R108, (**c**) Primer 2 and (**d**) OPQ6, of *Aspergillus fumigatus* strains (AF_S_, initial susceptible strain, AF_R0_, resistant induced strain, and AF_R1_, resistant strain after 30 days without antifungal) obtained during in vitro induction assay. AF_C_ represents a distinct *A. fumigatus* clinical strain, with a different pattern. 100 bp DNA ladder.

Dynamics of in vitro acquisition of resistance by *A. fumigatus* exposed to AFG is detailed in Table 1. After 30 days of exposure, resistance to AFG and cross-resistance to caspofungin (CAS) and micafungin (MFG) was developed. Exposure to AFG triggered macroscopic modification of morphology of *A. fumigatus* colonies, changing from the original green blue color to white (Fig. 1), becoming notably smaller. Microscopy showed absence of conidiation (data not shown). AF_R0_ and AF_R1_ showed the same macroscopic and microscopic phenotype. Similar changes have been reported in *A. fumigatus* exposed to antifungals during long periods^8–10^. RAPD analysis exhibits high discriminatory power for analysis of *A. fumigatus* strains when using this set of primers^11^. RAPD patterns obtained were 100% identical for the three strains (Fig. 2). A point mutation was found in AF_R0_ corresponding to replacement of glutamine by glutamate at position 671 of Fks1p (E671Q); similar mutation was found in AF_R1_. Since the resistant phenotype emerged abruptly, remaining stable following antifungal removal, it is highly plausible that this hot spot *FKS1* mutation E671Q might be responsible for the reduced susceptibility of AF_R0_ and AF_R1_. A mutation in *A. fumigatus FKS1* gene with potential to reduce echinocandin susceptibility is hereby described. Such mutation was never reported among *Candida* spp. An S678P amino acid change, equivalent to a mutation found in a resistant *Candida* isolate was described in a laboratory mutant of *A. fumigatus* and associated with resistance to CAS^12,13^. A mutation resulting in a F675S amino acid change was found in a chronic pulmonary aspergillosis isolate from a patient in whom micafungin treatment failed^10^. Point mutations in *FKS*1 genes are the main mechanism that is implicated in decreased echinocandin susceptibility, however, Arendrup and colleagues found no mutations in *FKS1* gene in two clinical isolates of *A. fumigatus* with MIC > 32 µg/mL to CAS^14^. Instead, an increased in *FKS1* gene expression was observed^14^. This mechanism may be implicated in tolerance to echinocandin therapy.

**Table 1:** Echinocandin Minimal Effective Concentration (MEC) values anidulafungin (AFG), caspofungin (CAS) and micafungin (MFG) distribution during in vitro induction assay with AFG of an *A. fumigatus* clinical isolate.

Induction day	MEC value (µg/mL)		
	AFG	CAS	MFG
0	≤ 0.015	≤ 0.015	≤ 0.015
5	≤ 0.015	0.06	≤ 0.015
10	≤ 0.015	0.125	0.03
15	≤ 0.015	0.125	0.06
20	≤ 0.015	0.125	0.125
25	0.03	0.25	0.125
30	> 8	> 8	> 8

**Figure 3:**
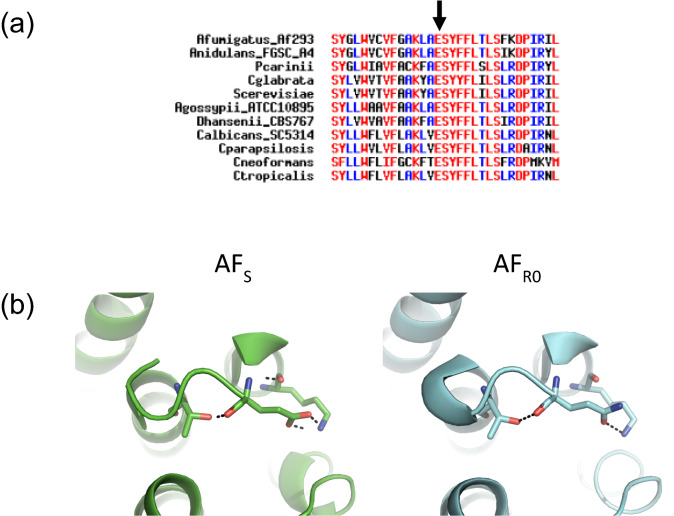
Sequence and structure analysis of the E671Q substitution in Fks1p. (**a**) Multiple protein sequence alignment of fungal Fks1 orthologues. A potential structural and/or functional role is suggested by the conservation of E671 even in distantly related species. (**b**) In the predicted structural model for this domain of the Fks1 protein (amino acids 400–900), the E671Q substitution would result in the loss of polar contacts with T677, disrupting the contacts between α-helices. AF_S_, the initial susceptible strain is shown in green, and AF_R0_, the resistant induced strain is shown in blue.

The E671Q mutation replaces an amino acid with a negatively charged side chain (glutamate) by an amino acid with a polar but uncharged side chain (glutamine). This position is conserved among several fungi (Fig. 3a). PROVEAN software considers an amino acid alteration at this position deleterious, suggesting that this region might have a relevant functional and/or structural role^15^. According to the three-dimensional (3D) structure obtained, E671 establishes polar contacts with K668 and T677 amino acids. Substitution by a glutamine would disrupt two of the three contacts with T677, which could distort conformation of the protein (Fig. 3b), given the proximity to a transmembrane domain (amino acids 679–699 in *S. cerevisiae*). Therefore, E671 may be necessary to maintain protein’s three-dimensional structure, supporting the assumption that such substitution could impair its function. Ultimately, two approaches might be taken: *FKS1* gene deletion in resistant strain to determine whether reversion to the susceptible phenotype occurs and site-directed mutagenesis in wild-type strain to observe whether resistant phenotype arises. Nevertheless, other mechanisms might also be involved in the development of echinocandin resistance, such as remodeling of cell wall components namely chitin levels, production of reactive oxygen species, alteration of the composition of plasma membrane lipids or expression levels of echinocandin target enzyme genes^16,17^.

Our results suggest that modification of Fks1p in *A. fumigatus* might confer echinocandins resistance. Given the emerging importance of clinical resistance among pathogenic fungi, it would be advisable to monitor the potential evolution of this mechanism in *Aspergillus* isolates from patients under echinocandin therapy.

## Material and methods

A suspension of 5 × 10^4^ conidia/mL of *A. fumigatus* (clinical brochoalveolar lavage isolate) was prepared in YEPD broth (0,3% yeast extract, 1% peptone, 2% dextrose) supplemented with sub-Minimal Effective Concentration (sub-MEC) (0.06 µg/mL) of AFG (Pfizer, Inc.) and incubated overnight, at 35 °C, 180 rpm. One mL was daily transferred to fresh YEPD supplemented with AFG. In parallel, 1 mL aliquot of was frozen (− 80 °C) but also cultured on YEPD agar at 35 °C for 72 h, to confirm viability and purity of culture. AFG concentration was increased to double whenever fungal growth was prominent, reaching a final concentration of 8 µg/mL. In vitro induction was carried out up to 30 days. Every 5 days, MEC values of the 3 echinocandin were determined according to CLSI^18^. A MEC value ≥ 1 µg/mL was considered resistance^19^. In order to assess the stability of echinocandin MEC values increments, the induced strain was daily sub-cultured for an additional 30 days in the absence of antifungal and MEC values re-determined. The resistant pattern remained stable. At the end of the assay, three strains were characterized: the initial susceptible strain (AF_S_), the induced strain (AF_R0_) and the strain obtained following additional 30 days without antifungal exposure (AF_R1_).

Genotyping by random amplification of polymorphic DNA (RAPD) of strains AF_S,_ AF_R0,_ AF_R1_ using primers R108 (5′-GTATTGCCCT-3′), OPAX (5′-AGTGCACACC-3′), OPQ6 (5′-GAGCGCCTTG-3′) and Primer 2 (5′-GCTGGTGG-3′) was performed^11^.

Following PCR with primers 5-GCTGAAGGATGTCGTCTGGA and 5-CGGCAAGTGATGGTCTCGTG, hot spot regions (between 1,875 and 4,318 bp) of *FKS1* gene (GenBank accession no. AFU79728) from AF_S_, AF_R0_ and AF_R1_ strains were amplified and sequenced were sequenced by Sanger method. The sequences were analyzed using BLAST Sequence Analysis Tool of NCBI.

The three-dimensional model for the Fks1 protein structure was obtained by modeling using the I-TASSER online server as previously described^20,21^. Structures were visualized in PYMOL v1.1r1.
